# Electron transport reactions of cyanobacterial photosynthesis and state-of-the-art in vivo measurement techniques

**DOI:** 10.1093/plphys/kiag107

**Published:** 2026-03-02

**Authors:** Laura T Wey, Peter R Bos, Michaela Crosbie, Pablo Ortega Martínez, Arjun Tiwari, Lauri Nikkanen

**Affiliations:** Molecular Plant Biology, Department of Life Technologies, University of Turku, Turku 20014, Finland; Molecular Plant Biology, Department of Life Technologies, University of Turku, Turku 20014, Finland; Molecular Plant Biology, Department of Life Technologies, University of Turku, Turku 20014, Finland; Molecular Plant Biology, Department of Life Technologies, University of Turku, Turku 20014, Finland; Molecular Plant Biology, Department of Life Technologies, University of Turku, Turku 20014, Finland; Molecular Plant Biology, Department of Life Technologies, University of Turku, Turku 20014, Finland

## Abstract

Cyanobacteria perform oxygenic photosynthesis using an integrated network of photosynthetic, respiratory, and auxiliary electron transport pathways embedded within the thylakoid membrane. Understanding how electrons are dynamically distributed among these interacting processes and how these flows are regulated under fluctuating environmental conditions requires approaches that can probe electron transport in vivo. In this review, we summarize the current understanding of linear, cyclic, auxiliary, respiratory, and extracellular electron transport in model cyanobacteria and highlight recent insights into the mechanisms that maintain redox balance and protect the photosynthetic apparatus. We critically assess state-of-the-art techniques used to quantify electron transport in vivo, including chlorophyll fluorescence, microscopy, membrane inlet mass spectrometry, differential absorbance spectroscopy, electrochromic shift measurements, photoelectrochemistry, and electron paramagnetic resonance spectroscopy. Finally, to address the major outstanding questions in regulation of photosynthesis, we recommend integration of techniques for simultaneous measurement of multiple processes and identify a need for non-invasive probes and modeling to achieve a systems-level understanding of cyanobacterial bioenergetics. Further study of nonmodel species is also needed to understand the diversity of cyanobacterial photosynthesis.

## Introduction

**Figure 1:**
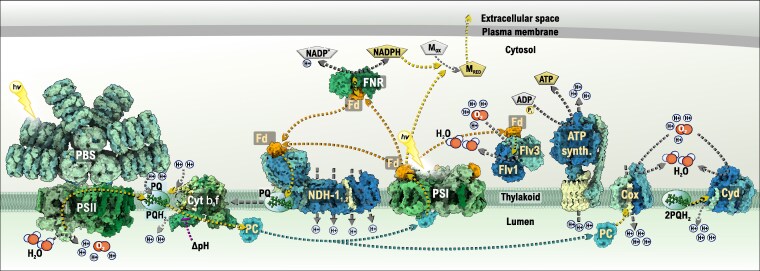
Photosynthetic electron transport routes in *Synechocystis* sp. PCC 6803. Schematic representations of either resolved or predicted structures of electron transport components are shown. As each Fd carries 1e^−^, 2Fd^−^ are needed for reduction of NADP^+^ by FNR, 4Fd^−^ for reduction of O_2_ by Flv1/3, and 2Fd^−^ for reduction of PQ to PQH_2_ by NDH-1, which also involves binding of 2H^+^ from the cytosol and active pumping of 4H^+^ to the lumen (2H^+^/e^−^). Both Cox and Cyd take up 4H^+^ from the cytosol to reduce O_2_ to 2H_2_O. This is coupled with the pumping of 4H^+^ (1H^+^/e^−^) to lumen by Cox. Cyd does not pump protons, but 4H^+^ are released to the lumen upon oxidation of 2PQH_2_ to provide 4e^−^ for the reduction of O_2_. Each 360^°^ rotation of ATP synthase produces 3ATP, but the number of H^+^ required depends on the number of monomers in the membrane-spanning c-ring, which varies between 13 and 15 in cyanobacterial species (Pogoryelov et al. 2007). *Synechocystis* has a c_14_ ring and requires 4.67H^+^/ATP. PBS = phycobilisome, structure based on Domínguez-Martín et al. (2022). PSII = photosystem II, dimeric structure from *Synechocystis* sp. PCC 6803 (Gisriel et al. 2022), PDB accession 7RCV. Cyt b_6_f = cytochrome b_6_f, dimeric structure from *Synechocystis* sp. PCC 6803 (Proctor et al. 2022), PDB accession 7ZXY. PC = plastocyanin, structure of reduced PC from *Synechocystis* sp. PCC 6803 (Bertini et al. 2001), PDB accession 1JXD. Cyt c_6_ = cytochrome c_6_, structure from *Synechococcus* sp. PCC 7002 (Bialek et al. 2014), PDB accession 4EID. PSI and Fd = photosystem I trimer with bound ferredoxins 1 from *Thermosynechococcus elongatus* (Li et al. 2022a), PDB accession 7FIX (The bound PC depicted in the figure is not a part of the original structure). FNR and Fd = ferredoxin-NADP ^+^ -oxidoreductase with bound ferredoxin from *Anabaena* PCC 7119 (Morales et al. 2000), PDB accession 1EWY. Flv1/Flv3/Fd = heterodimeric structure of *Synechocystis* sp. PCC 6803 Flv1 and Flv3 with bound ferredoxin 1 predicted with Alphafold2-Multimer (no structures of the full length Flv proteins have been resolved). NDH-1 and Fd = NAD(P)H-dehydrogenase-like complex 1_1_ (NDH-1L) with bound ferredoxin 1 from *Thermosynechococcus vestitus* BP-1 (Zhang et al. 2020), PDB accession 6L7O. ATP synth. = CF_1_F_O_ ATP synthase from *Spinacia oleracea* (Hahn et al. 2018), PDB accession 6FKF. Cox = cytochrome c oxidase from *Paracoccus denitrificans* (Kolbe et al. 2021), PDB accession 7AU3. Cyd = cytochrome bd quinol oxidase from *Escherichia coli* (Safarian et al. 2019), PDB accession 6RKO. Cox and Cyd structures in cyanobacteria may differ from the structures shown, which are used here for illustrative purposes. M = endogenous electron mediator that facilitates exoelectrogenesis.

The photosynthetic electron transport chain (PETC) (Fig. 1) converts solar energy into chemical energy, driving the fixation of inorganic carbon. In most cyanobacteria, except for *Gloeobacter* species as well as the more recently described *Anthocerotibacter panamensis*, electron transport occurs across the thylakoid membrane (Mareš et al. 2019; Rahmatpour et al. 2021). The cyanobacterial thylakoid membrane houses components of both photosynthetic and respiratory electron transport, enabling close coordination between light-driven (linear, cyclic, and auxiliary) and respiratory electron transport pathways. Much of this has been elucidated in detail for the model cyanobacterium *Synechocystis* sp. PCC 6803, and other model species, such as *Synechococcus elongatus* PCC 7942 and filamentous cyanobacteria *Anabaena* sp. PCC 7120 (see reviews by Lea-Smith et al. 2016; Lea-Smith and Hanke 2021; Burnap 2023; Shimakawa 2023). Here we give an overview of our latest understanding of photosynthetic and other electron transport pathways and highlight several outstanding questions for model and especially emerging model species, such as *Synechococcus* sp. PCC 11901 (Gale et al. 2019; Mills et al. 2022). We then describe the state-of-the-art techniques that can be harnessed to study these electron transfer processes in vivo and hopefully answer these outstanding questions.

### Linear electron transport

Linear electron transport (LET) begins with light harvesting and charge separation at PSII, a ∼700-kDa homodimeric complex surrounded by phycobilisome antennae (Domínguez-Martín et al. 2022; Gisriel et al. 2022). Excitation energy is funneled from the phycobilisome to the P680 special-pair chlorophylls of the PSII reaction center by Förster resonance energy transfer as well as delocalization of excitons between closely coupled pigments (Sil et al. 2022; Sohoni et al. 2023). Excited P680* donates 1 electron to pheophytin (Pheo), generating the powerful oxidant P680^+^. This oxidizing power drives water splitting at the Mn_4_CaO_5_ cluster of the oxygen evolving complex (OEC) on the lumenal side, releasing O_2_ and protons into the lumen, along with electrons that re-reduce P680^+^ to its ground state with each photochemical turnover. Within the PSII complex, the electron passed from P680* to Pheo^−^ is transferred sequentially to Q_A_ and then to Q_B_. Two Q_A_→Q_B_ transfers, accompanied by the uptake of 2 cytosolic protons, are required to reduce 1 plastoquinone (PQ) to plastoquinol (PQH_2_). For a recent review on PSII, see (Shevela et al. 2023).

PQH_2_ is released from the Q_B_ binding pocket and diffuses through the membrane to the cytochrome *b_6_f* (Cyt *b_6_f*) complex, a dimeric complex containing 2 quinone-binding sites (Q_p_ and Q_n_) per monomer (Proctor et al. 2022). PQH_2_ oxidation occurs at the lumenal Q_p_ site, where 2 protons are released to the lumen and 2 electrons are liberated. One electron is transferred via the Rieske 2Fe-2S protein and haem f in the high-potential chain to plastocyanin (PC), a small, copper-containing protein diffusible in the lumen that then donates the electron to oxidized P700^+^ in PSI. The other electron is recycled in the low-potential chain to reduce PQ at the Q_n_ site, drawing 2 additional cytosolic protons to complete the Q-cycle (Mitchell 1975; Malone et al. 2021). Under copper limitation, cytochrome c_6_ fulfils the role of PC (Durán et al. 2004).

In most cyanobacteria, including *Synechocystis*, PSI forms a trimeric complex comprising multiple protein subunits and cofactors (Malavath et al. 2018; Li et al. 2022a) but can form tetramers in species such as *Chroococcidiopsis* TS-821 (Semchonok et al. 2022) and in filamentous *Anabaena*, particularly in response to high light exposure (Li et al. 2019). Excitation energy is harvested by PSI-associated chlorophylls and transferred from phycobilisomes, either via state transitions, spillover, or direct PBS–PSI coupling observed in several cyanobacterial species (Akhtar et al. 2022; Shimizu et al. 2023; van Stokkum et al. 2025), and funneled to the P700 special-pair chlorophylls, forming the excited state P700*, one of nature's most powerful biological reductants (van Stokkum et al. 2025). Each excited P700* donates a single electron sequentially to the primary acceptor chlorophyll A_0_, which transfers it to phylloquinone A_1_, then to the iron–sulfur clusters F_X_, F_A_, and F_B_ (Caspy et al. 2020). The terminal electron acceptor is the soluble protein ferredoxin (Fd), or under Fe-limitation or saline stress, its functional analogue flavodoxin (Hagemann et al. 1999). Reduced Fd transfers the electron to ferredoxin–NADP^+^ reductase (FNR), which catalyzes the reduction of NADP^+^ to NADPH, converting light energy into reducing power for CO_2_ fixation in the Calvin–Benson–Bassham cycle and other anabolic pathways. FNR has been proposed to bind to phycobilisome rods in cyanobacteria (Liu et al. 2019; Zheng et al. 2021, 2023), but while the phycobilisome binding has been suggested to be required for photoheterotrophic growth (Li et al. 2022b), the physiological significance of it requires further investigation.

Fd functions as a electron distribution hub to various other processes in the cell as well, including cyclic and auxiliary pathways (see below), the thioredoxin system, nitrogen assimilation, and the bidirectional [NiFe] hydrogenase (Hox) (Cassier-Chauvat and Chauvat 2014; Mondal and Bruce 2018; Artz et al. 2020). Exactly how the distribution of electrons from Fd to the various acceptors is determined and what the physiological roles and levels of redundancy are between the 11 Fd isoforms of *Synechocystis* (Artz et al. 2020; Nikkanen et al. 2025) remain topics of ongoing investigation. Nonetheless, it was recently reported that in conditions where substrate (NADP^+^) availability does not limit the activity of FNR, it is the main acceptor of electrons from photosynthetically reduced Fd, outcompeting auxiliary pathways (Hubáček et al. 2024).

Electron transport in the PETC is coupled to proton translocation across the thylakoid membrane, from the n (negative) side (the cytoplasm in cyanobacteria) to the p (positive) side (the lumen): 4H^+^ are released upon oxidation of 2H_2_O at the OEC, 2H^+^ are bound from the cytosol upon reduction of PQ at the Q_B_ site, and 4H^+^ are released from oxidation of 2PQH_2_ at the Q_p_ site of Cyt b_6_*f* in the Q cycle, while 2H^+^ are bound from the cytosol to reduce PQ at the Q_n_ site. The established proton gradient (ΔpH) is further increased by consumption of protons in the cytosol, most importantly by carbon fixation as well as water-water cycles, and by proton pumping in cyclic electron transport. The ΔpH together with the electric field (ΔΨ) generated by charge separation at the photosystems and differing concentrations of ions such as K^+^, Cl^−^, Mg^2+^, Ca^2+^, and Mn^2+^ on the 2 sides of the thylakoid membrane, constitutes the proton motive force (*pmf*). The *pmf* powers ATP synthase, which catalyzes the formation of ATP from ADP and inorganic phosphate.

### Cyclic electron transport

When ATP demand exceeds NADPH requirements or redox homeostasis is perturbed, electrons are rerouted from Fd back to the PQ pool via cyclic electron transport (CET). While other pathways have been proposed for chloroplasts, mainly the so-called antimycin A-sensitive CET pathway dependent on the presence of the PGRL1 and PGR5 proteins and catalyzed by a hypothetical enzyme ferredoxin-plastoquinone reductase (FQR), (Munekage et al. 2002; DalCorso et al. 2008; Yamamoto and Shikanai 2019), in cyanobacteria CET is most likely exclusively catalyzed by the NADH-dehydrogenase-like complex (NDH-1), which is also known as Photosynthetic Complex I. CET-mediated ΔpH generation is, at least in standard growth conditions, fully dependent on NDH-1 (Miller et al. 2021), and the knockout mutant of the *Synechocystis* homolog of PGR5 shows no impairment of CET (Nikkanen et al. 2024). Instead, cyanobacterial PGR5 homologs may have a role in regulation of the *pmf* in high light (Nikkanen et al. 2024). However, potential other CET pathways functioning in specific environmental conditions cannot be ruled out.

Four forms of NDH-1 exist in cyanobacteria: NDH-1_1_ and NDH-1_2_ involved in respiration and cyclic electron transport, while NDH-1_3_ and NDH-1_4_ function in CO_2_ uptake by coupling proton pumping and PQ reduction to carbonic anhydrase activity (Ohkawa et al. 2000; Peltier et al. 2016; Zhang et al. 2025). NDH-1_1_ and NDH-1_2_ operate by coupling PQ reduction to proton pumping with a 2H^+^/e^−^ stoichiometry (as inferred from homology with respiratory Complex I while direct experimental evidence is still lacking) to increase generation of ATP and thereby the ATP/NADPH ratio (Schuller et al. 2019, 2020). NDH-1_3_ and NDH-1_4_, however, lack a fourth H^+^ release module and probably only release 3H^+^ to the lumen for each PQ reduced (1.5H^+^/e^−^) (Zhang et al. 2025). The increased acidification of the lumen by CET is also instrumental in inducing the photoprotective mechanism of photosynthetic control (see below). CET activity is modulated by NdhO-mediated inhibition (Zhang et al. 2020), while presence of NdhV promotes higher NDH-1 activity (Chen et al. 2016). Moreover, it is affected by feedback from the cellular ATP/ADP ratio, with high ATP levels down-regulating CET to prevent overproduction (Fisher et al. 2019).

### Auxiliary and respiratory electron transport

Flavodiiron proteins (Flvs) act as rapid photoprotective electron sinks in cyanobacteria. *Synechocystis* has 4 isoforms (Flv1-4), which form hetero-oligomers (Flv1/Flv3 and Flv2/Flv4) that accept electrons from reduced Fd1 downstream of PSI and reduce O_2_ to H_2_O (Allahverdiyeva et al. 2013; Santana-Sánchez et al. 2019; Nikkanen et al. 2020, 2025; Sétif et al. 2020). This “Mehler-like” reaction bypasses NADPH formation and mitigates PSI over-reduction and ensuing photodamage, especially under fluctuating or high light conditions (Allahverdiyeva et al. 2013). PSI can still be reduced if electron supply from the donor side exceeds the kinetic capacity of Flvs and other acceptor side sinks. For example, activation of Flvs upon dark-to-light transitions is not instant but occurs after c.a. 500 ms of light, resulting in transient reduction of P700 and ferredoxin in *Synechocystis* (Nikkanen et al. 2020). It was recently reported that Flv1/3 and Flv2/4 activity is modulated by pH-dependent reversible association with the thylakoid membrane, with alkalization of the cytosol resulting in Flv heterodimers being repelled from the membrane and decreased activity, and low pH promoting membrane attachment and higher activity (Nikkanen et al. 2025). Disulfide-dependent formation of heterotetramers was reported for Flvs in the moss *Physcomitrium patens* (Beraldo et al. 2024), but whether a similar level of regulation also exists in cyanobacteria remains an open question.

Respiratory electron transport (RET) introduces electrons into the PQ pool, which is shared with the photosynthetic LET and CET pathways, via NDH-1, NDH-2, and succinate dehydrogenase (SDH) (Cooley and Vermaas 2001). The main source of electrons for RET is likely NADPH produced in the oxidative pentose phosphate (OPP) pathway (Kusama et al. 2022b), which can be used by the small isoform of FNR to produce reduced Fd to drive NDH-1 activity in RET or CET (Miller et al. 2022). Alternatively, reduced Fd can be generated from decarboxylation of pyruvate by pyruvate:ferredoxin oxidoreductase (PFOR) especially under photomixotrophy (Wang et al. 2022). In RET electrons are ultimately used for O_2_ reduction by the respiratory terminal oxidases (RTOs) aa_3_-type cytochrome-c oxidase (COX), cytochrome bd quinol oxidase (Cyd), or the alternative respiratory terminal oxidase (ARTO) on the cytoplasmic membrane, which harbors a partial RET chain (Ermakova et al. 2016; Shimakawa et al. 2020a). Some cyanobacteria, such the filamentous *Anabaena* sp. PCC 7120 also have homologs of the plastid terminal oxidase PTOX (McDonald et al. 2003). RTOs have been shown to be important for efficient growth under changing light intensities and act as a minor photoprotective sink for excessive electrons in the PETC (Lea-Smith et al. 2013; Ermakova et al. 2016). In phototrophic, dark-adapted WT *Synechocystis* cells the respiration rate, measured as O_2_ uptake in dark, is typically 3 to 8 µmol O_2_ mg Chl^−1^ h^−1^ (Ermakova et al. 2016; Shimakawa et al. 2020a; Nikkanen et al. 2025), and can increase to ∼20 µmol O_2_ mg Chl^−1^ h^−1^ upon addition of an organic carbon source (Shimakawa et al. 2020a; Ortega-Martínez et al. 2024), or transiently up to ∼50 µmol O_2_ mg Chl^−1^ h^−1^ after light adaption (Shimakawa et al. 2020a).

### Exoelectrogenesis

Cyanobacteria can also export electrons to their environment via exoelectrogenesis (also called extracellular electron transfer) (Wey et al. 2019; Zhu et al. 2023). Under illumination, the majority of electrons eventually exported originate from water oxidation by PSII (Yagishita et al. 1993; Pisciotta et al. 2010; Bombelli et al. 2011), with some contribution from intertwined respiration (Saper et al. 2018). The addition of glucose transiently increases exoelectrogenesis (Yagishita et al. 1997; Saper et al. 2018), and under darkness the current relates to glycogen levels (Tanaka et al. 1985; Yagishita et al. 1993). Studies that used inhibitors of components of the PETC downstream of PSII (that unfortunately often participate in electrochemical side-reactions with electrodes) point toward electron exit downstream of PSI, such as at Fd or NADPH (Yagishita et al. 1993; Pisciotta et al. 2010; Bombelli et al. 2011). Perhaps counterintuitively, NADPH levels produced from the OPP pathway in the dark are critical for exoelectrogenesis in the light (Hatano et al. 2022).

The balance between intracellular and extracellular electron sinks remains to be fully elucidated, predominantly due to lack of analytical tools employed under physiological conditions. The thylakoid-localized RTOs COX and Cyd are in competition with exoelectrogenesis in the dark, and Flvs are in competition with exoelectrogenesis in the light, from experiments with artificial electron acceptors (Bradley et al. 2013; Saar et al. 2018; Sekar et al. 2018; Yuan et al. 2025). Exoelectrogenesis in the light is abolished by inhibition of the CBB cycle by glycolaldehyde (Kusama et al. 2022a).

The bioelectrochemical mechanism of extracellular electron transfer in *Synechocystis* is not direct via, for example, type IV pili or redox proteins embedded in the extracellular matrix but indirect electron transfer facilitated by an endogenous, diffusible redox mediator (Saper et al. 2018; Thirumurthy et al. 2020; Wey et al. 2021, 2024; Kusama et al. 2022a), whose identity (or identities) remains an open question for consensus due to a variety of bespoke electrochemical set-ups and experimental conditions. NADPH has been both identified and excluded to shuttle the electrons outside the cell and to an electrode (Shlosberg et al. 2021; Kusama et al. 2022a; Wroe et al. 2024).

The physiological role of exoelectrogenesis remains unclear. Exoelectrogenesis facilitates reductive metal acquisition in some species, as *Synechococcus elongatus* PCC7942 shows increased ferricyanide reduction under iron-limited conditions (Gonzalez-Aravena et al. 2018). Exoelectrogenesis is most commonly proposed to function in photoprotection by alleviating over-reduction of the photosynthetic electron transport chain, although direct evidence for this is lacking. Exoelectrogenesis could also play a role in cell-to-cell signaling, based on the presence of diffusible redox mediators in the extracellular space.

The possible magnitude of exoelectrogenesis has been estimated to be 40% of photosynthetically derived electrons, but this relies on assumptions about essential metabolic demands and export efficiency that are poorly constrained for photosynthetic bacteria and instead extrapolated from nonphotosynthetic electrogens (McCormick et al. 2015; Howe and Bombelli 2020). Recent photoelectrochemical measurements without artificial mediators report a low external quantum efficiency (∼0.3%) (Chen et al. 2022), suggesting that exoelectrogenesis in natural systems is likely much lower than theoretical maxima.

### Feedback regulation of photosynthetic electron transport

When the electron transport rate exceeds the electron sink capacity of downstream metabolism, such as under high or fluctuating light conditions, excessive reduction of PSI results in production of reactive oxygen species (ROS) and damage to F_X_, F_B_, and F_A_ clusters of PSI (Tiwari et al. 2016, 2024). Generation of sufficient *pmf* is essential to prevent this damage. While both components of *pmf*, ΔpH and Δψ, are thermodynamically equal in driving ATP synthesis (Mitchell 1966), inhibition of PQH_2_ oxidation at the Cyt b_6_*f* in a process known as photosynthetic control specifically depends on acidification of the lumen (Degen and Johnson 2024). In algae and plant chloroplasts, a P173L mutation in the Rieske subunit of Cyt b_6_*f* activates photosynthetic control even under more alkaline conditions (Ozawa et al. 2023), but similar studies in cyanobacteria have not been conducted. It has also been suggested that reduction of a conserved disulfide in the Rieske protein by a thioredoxin could raise the threshold lumenal pH that allows deprotonation of PQH_2_ at the Q_p_ site, providing an additional layer of regulation (Hald et al. 2008; Degen and Johnson 2024). Experimental evidence for such regulation is, however, lacking.

In plants and algae, acidification of the thylakoid lumen is also a prerequisite for induction of nonphotochemical quenching (NPQ), the dissipation of excessive excitation energy that cannot be used for photochemistry as heat (Krishnan-Schmieden et al. 2021). In cyanobacteria, however, the induction of NPQ is independent of ΔpH and requires instead a light-dependent conformational change in the orange carotenoid protein (OCP) (García-Oneto et al. 2024). The binding of the OCP to the phycobilisome, and thus induction of NPQ, is facilitated by light-intensity–dependent changes in conformational positions of the phycobilisome rods (Domínguez-Martín et al. 2022). Sudden spikes in Δψ, for example, during rapid fluctuations in light intensity, cause generation of highly reactive singlet oxygen (^1^O_2_) in charge recombination reactions, damaging PSII (Davis et al. 2016). This makes it vital for photosynthetic organisms to dynamically adjust the composition of the *pmf* to avoid the Δψ spikes and damage to the photosynthetic apparatus (see Box 1).

Box 1 Regulation of magnitude and composition of the *pmf* Adjusting the *pmf* and its composition of ΔpH and Δψ is achieved by regulating the conductivity of ATP synthase to protons and by orchestrating activities of ion channels, transporters, and exchangers on the thylakoid membrane. Cyanobacterial ATP synthase lacks the disulfide switch in the F_1_γ subunit whose reduction by thioredoxin activates the chloroplast ATP synthase upon illumination (Hisabori et al. 2013). Instead, it is regulated at least by inhibitory actions of the F_1_ε subunit (Imashimizu et al. 2011; Murakami et al. 2018) and the recently identified AtpΘ protein (Song et al. 2022), which both prevent ATP hydrolysis in dark or under low *pmf*. Metabolic control of ATP synthase activity through substrate availability as well as regulation induced by CO_2_ concentration are well-established (Kohzuma et al. 2013). Moreover, the *Synechocystis* PGR5 homolog Ssr2016 (Yeremenko et al. 2005) has been suggested to be involved in downregulation of ATP synthase conductivity under high irradiance (Nikkanen et al. 2024).

In plants and algae chloroplast, thylakoid ion transporters include the K^+^/H^+^ exchanger KEA3 (Armbruster et al. 2014; von Bismarck et al. 2023), voltage-gated Cl^−^ channel VCCN1 (Herdean et al. 2016b), and the Cl^−^ channel CLCe (Herdean et al. 2016a). For a recent review on thylakoid ion transport, see (Kunz et al. 2024). In cyanobacteria, only a single K^+^ channel (SynK) has so far been identified in thylakoids (Zanetti et al. 2010). *Synechocystis* cells lacking SynK grow poorly under high light and display impaired generation of ΔpH and lower electron transport rate (Checchetto et al. 2012). Additionally, Na^+^/H^+^ antiporters NhaS1 and NhaS3 as well as the putative Ca^2+^/H^+^ exchanger SynCAX have been suggested to also localize to the thylakoid membrane (Baers et al. 2019). Nonetheless, further research on regulation of thylakoid ion transport is required.

Another form of feedback regulation of photosynthetic electron transport is achieved by state transitions. Preferential excitation of either of the two photosystems by different wavelengths of light can result in a redox imbalance of the PETC and production of ROS (Mattila et al. 2020). To counter this, changes in the redox state of the PQ pool trigger a change in the tendency of the phycobilisome antennae to transfer excitation energy to either PSII or PSI. These changes are known as state transitions. While in plants and algae the role of the STT7/STN7 kinase in triggering state transitions through phosphorylation of LHCII proteins is well studied, in cyanobacteria phosphorylation reactions are not involved and the molecular mechanism of induction remains unclear (Calzadilla et al. 2019; Calzadilla and Kirilovsky 2020; Bhatti et al. 2021; van Stokkum et al. 2025). It was shown, however, that cyanobacterial state transitions are mostly defined by quenching of phycobilisomes and PSII and not by antenna movement (Bhatti et al. 2021).

## In vivo measurement techniques

### Chlorophyll fluorescence

Chlorophyll fluorescence has been of paramount importance in the study of photosynthesis (Krause and Weis 1991; Murchie and Lawson 2013). For example, pulse amplitude modulation (PAM) fluorometry uses short non-actinic (nonphotosynthesis-inducing) light pulses to probe the reaction center state of mostly PSII. The open reaction center state (F_o_) results in a lower fluorescence signal than the fully closed state (F_m_) and the ratio of the two (F_v_/F_m_) has been used as a probe for plant fitness, photosynthetic capacity, and photosynthetic quantum yield. However, these parameters and methods of determining them in cyanobacteria are problematic and require caution (Ogawa et al. 2017; Garab et al. 2023). A major source of error is caused by the intertwined pathways of respiration and photosynthesis in cyanobacteria (Ogawa et al. 2017). PQ is used in both pathways, resulting in only partial oxidation of the PQ pool in darkness, which increases F_o_. Moreover, although thylakoid microdomains with enriched PSII or PSI have been reported, cyanobacterial thylakoids lack the strict lateral heterogeneity of PSII and PSI localization seen in chloroplasts (Strašková et al. 2019; Bhatti et al. 2021; Huokko et al. 2021; Gu et al. 2022; Kaňa et al. 2023; Bos et al. 2025) and have a much higher PSI/PSII ratio (Moore and Vermaas 2024). This results in an increased amount of excitation spillover between the photosystems taking place in comparison to plants or algae. It was recently reported that up to 40% of excitation energy at PSII can be lost as spillover to PSI in isolated cyanobacterial thylakoid membranes and up to 20% in intact cells (Akhtar et al. 2024). Background fluorescence (F_o_) is further increased by phycobilisomes, both connected and unconnected. Phycobilins have a higher intrinsic fluorescence yield than chlorophylls and energy transfer from phycobilins to the PSII reaction center is largely irreversible, resulting in a high phycobolin-derived fluorescence signal that is mostly unresponsive to closure of PSII reaction centres (Joshua et al. 2005; Acuña et al. 2016). This issue is exacerbated by instrumental factors, as the standard red (620 nm) measuring light used in many PAM fluorometers efficiently excites phycocyanin and further increases phycobilin fluorescence. These factors lead to substantial underestimation of PSII quantum yield. The problem can be alleviated to some extent by using a blue measuring light (460 nm) that is weakly absorbed by phycobilins (Acuña et al. 2016). However, PSI fluorescence remains an issue and requires adaptation of measurement protocols designed for plants to induce state I (PBS association with PSII) and correction for PSI fluorescence (Ogawa and Sonoike 2016; Bhatti et al. 2021). Addition of DCMU is considered a necessity for determining F_m_ (Stirbet et al. 2019). These factors make PAM fluorometry a less robust method for determination of cyanobacterial photosynthetic efficiency in comparison to plants. Finally, the observation that a light-adapted charge-separated population of PSII reaction centers contribute to variable chlorophyll fluorescence and thereby results in further underestimation of PSII quantum yield during illumination (Sipka et al. 2021) is important to bear in mind when interpreting photosynthetic parameters derived from PAM fluorometry.

The J state in the OJIP induction curve (named after the four stages or deflection points of the curve) has been shown to represent a semi-quantitative measure for the reduction state of the PQ pool (Zavřel et al. 2026). With inhibitors of the electron transport chain, Vj was shown to present an accurate estimate of the PQ pool redox state. However, it should be noted that the interpretation of the OJIP curve in plants is controversial, since at least four different ways to assign kinetic features of the curve to electron transfer components have been proposed (Lazár 2003; Kalaji et al. 2014, 2017; Stirbet and Govindjee 2016; Zavafer et al. 2020). How the curve should be interpreted in cyanobacteria remains to be elucidated.

### Microscopy

Several imaging methods have been applied in cyanobacteria to investigate the localization and energetic connection of PSI and PSII, most notably in *Synechocystis* and *Synechococcus elongatus* (Casella et al. 2017; MacGregor-Chatwin et al. 2017, 2022). However, the imaging techniques with the highest resolution, electron microscopy (EM) and atomic force microscopy (AFM), require fixation or isolation of the thylakoid membrane, making them unsuitable for studying the in vivo dynamics (Demir-Yilmaz et al. 2021; Wietrzynski et al. 2025). Despite the obvious disadvantage of a decreased resolution, several light microscopy techniques have been applied in cyanobacterial cells. Light microscopy allows in vivo imaging and offers a spectral range that can be utilized for simultaneous localization of multiple fluorophores (Grigoryeva 2020). For example, a combination of preferential excitation and emission and YFP tagging of PSI enabled simultaneous visualization of PSI, PSII, and phycobilisomes. However, low fluorescence intensity of some fluorophores and a poor z-resolution might complicate interpretation.

In the future, research on cyanobacterial photosynthesis can benefit greatly from the introduction of fluorescent probes that are sensitive to pH, membrane potential, MgATP^2−^, NADH/NAD^+^, and cellular thiol redox state, as has been shown in plants (Schwarzländer et al. 2008, 2012; Lim et al. 2020, 2022; Steinbeck et al. 2020; Zheng et al. 2025). The physiological state of plant cells was measured live upon light induction on cell level. Similar approaches could generate significant insights in cyanobacterial research, although the small cell size and decreased spectral range might complicate the introduction of fluorescent probes in cyanobacteria.

### Measurement of photosynthetic gas fluxes

Measurement of oxygen evolution provides a direct way to quantify PSII photochemistry in cyanobacterial cultures. This can be done by Clark-type O_2_ electrodes, which can provide reliable estimates of net O_2_ evolution (gross minus uptake), or by membrane inlet mass spectrometry (MIMS). MIMS is a powerful analytical technique that enables real-time quantification of fluxes of volatile compounds, including gases such as O_2_, CO_2_, H_2_, and N_2_ (Beckmann et al. 2009; Burlacot et al. 2020). The MIMS setup consists of a closed measurement chamber coupled via a gas-permeable membrane (silicone or polytetrafluoroethylene [Teflon] membranes are often used) to a high-vacuum inlet mass spectrometer (Burlacot et al. 2020). Calibration against known gas standards ensures quantitative accuracy, while a fast response time permits kinetic analyses of rapid metabolic transitions. Dissolved gases diffuse across the membrane to the vacuum of the MS and are detected by their characteristic mass-to-charge ratios, allowing isotopic discrimination. The different atomic weight of isotopes, such as ^16^O_2_ and ^18^O_2_, or ^12^CO_2_ and ^13^CO_2_, allows dissection of molecular mechanisms of photosynthetic gases exchange, including precise kinetics of O_2_ production at the OEC (Shevela and Messinger 2013) and distinguishing O_2_ consumption due to respiration or the Mehler-like reaction from O_2_ evolution (Ermakova et al. 2016; Mustila et al. 2016; Santana-Sánchez et al. 2019).

For inorganic carbon (C_i_), MIMS enables direct measurement of CO_2_ uptake and fixation, as well as the activity of the cyanobacterial CO_2_-concentrating mechanism (CCM) (Liran et al. 2018; Douchi et al. 2019; Santana-Sánchez et al. 2023). MIMS was also used to clarify the role of carbonic anhydrases in maintaining intracellular C_i_ pools (Douchi et al. 2019), and how bicarbonate interacts with PSII (Shevela et al. 2020). Beyond O_2_ and CO_2_, MIMS can monitor additional gases of physiological relevance, including H_2_ evolution, N_2_ fixation, N_2_O emission (Cournac et al. 2004; Kosourov et al. 2021; Santana-Sánchez et al. 2023).

Despite its versatility, MIMS has several technical and practical constraints. Baseline drift and signal-to-noise issues limit reliable quantification of very low fluxes. Accurate operation requires taking into account membrane/MS gas consumption and careful calibration with gas-saturated water standards (O_2_, CO_2_, N_2_, Ar) and isotope-labeled standards (^18^O_2_, ^13^CO_2_, ^15^N_2_), which must be repeated frequently due to membrane aging and fouling. Because gas diffusion across the membrane occurs at a time scale of seconds, MIMS cannot resolve very fast kinetics, in contrast to spectroscopic approaches operating at nanosecond-to-millisecond resolution. A further practical limitation is that most instruments are custom-built, requiring expertise in vacuum systems and mass spectrometer maintenance and possibly decreasing reproducibility across laboratories (Burlacot et al. 2020).

### Differential absorbance spectroscopy

Differential absorbance spectroscopy in the near-infrared (NIR) region has become a key tool for studying in vivo electron transport kinetics and redox dynamics around PSI. In conventional measurements of P700 oxidation based on absorbance spectroscopy (eg with the Dual-PAM), redox-dependent absorbance changes of PC, P700, and Fd are confounded due to spectral overlap of their redox signals. The Dual KLAS-NIR spectrophotometer (Walz) (DKN), however, enables their deconvolution after determination of the distinct near-infrared (NIR) absorption signatures of each component. DKN allows measurement of four wavelength difference signals in the NIR regions (785 to 840 nm, 810 to 870 nm, 870 to 970 nm, and 795 to 970 nm) to isolate cofactor-specific redox signals. These isolated signals are fitted to differential model plots (DMPs) to deconvolute the overlapping signals and assign the proportions of each component's contributions at each wavelength pair (Klughammer and Schreiber 2016). DMPs must be empirically obtained for each component in conditions where the redox changes are predominantly observed in only one of them and need to be measured separately for different species (Klughammer and Schreiber 2016; Sétif et al. 2019). This approach allows extraction of individual kinetic traces of PC, P700, and Fd redox kinetics in vivo. In addition, the relative pool sizes of the cofactors can be inferred from maximal absorption changes. Contributions of cytochrome c_6_ to these redox changes have also been explored (Shimakawa et al. 2020b), and the technique has been used to examine electron transfer in cyanobacteria from Fd to its acceptors such as FNR and NDH-1 and to identify Fd1 as the main electron donor to the Mehler-like reaction catalyzed by Flv1/3 and Flv2/4 heterodimers (Nikkanen et al. 2020, 2025; Sétif et al. 2020; Hubáček et al. 2024).

Despite its strengths, differential NIR spectroscopy has some limitations. The Fd signal typically has a small amplitude and rapid reoxidation of Fd in cyanobacteria, due to efficient electron transfer to O_2_ by Flv proteins, making measurement of accurate DMPs challenging. This can be addressed either by determining the Fd model spectra under anoxic conditions (Theune et al. 2021) or by using Flv deletion strains (Nikkanen et al. 2020).

Among differential spectroscopy approaches, Joliot-Type Spectrophotometers (JTS, Spectrologix/BioLogic) have been widely used to probe redox kinetics of multiple cofactors, such as those of cyt b_6_*f*. The JTS is a pump-probe spectrophotometer that uses low-intensity measuring pulses either from monochromatic LEDs or through specific interference filters, while being weak enough to avoid actinic effects. The latest JTS-150 can measure up to nine wavelengths in real time with microsecond resolution, allowing simultaneous tracking of multiple cofactors. Cyt *f* redox kinetics are typically monitored at 554 nm and b hemes at 563 nm, with baseline corrections between 546 and 574 nm. Measurement of Cyt b*_6_f* redox changes has been used in cyanobacteria to examine electron transfer kinetics in the chlorophyll d-containing cyanobacterium *Acaryochloris marina* (Bailleul et al. 2008) and in the fast-growing strain *Synechococcus elongatus* UTEX 2973 (Ungerer et al. 2018), upon addition of glucose as an organic carbon source in *Synechocystis* (Solymosi et al. 2020; Ortega-Martínez et al. 2024), or in the presence of a strong exogenous electron sink (Hubáček et al. 2024).

### Electrochromic shift

Quantifying the magnitude of the *pmf* and balance of the ΔΨ and ΔpH components is central to understanding photosynthetic regulation and energy conversion efficiency. Measurement of the electrochromic shift (ECS) is a versatile and non-invasive spectroscopic tool that enables real-time analysis of *pmf* dynamics in vivo (Bailleul et al. 2010). The ECS signal arises from the phenomenon known as the Stark effect, where absorption spectra of pigments such as carotenoids undergo a shift in the presence of an electric field. By determining this spectral shift of pigments embedded in the thylakoid membrane, the electric field generated by photosynthetic charge separations can be quantified by absorbance spectroscopy (Witt 1979).

The ECS spectra differs between photosynthetic organisms due to differences in pigment composition, making determination of species-specific spectra essential (see Box 2). Cyanobacterial ECS, measured as 500 and 480 nm absorbance difference (Viola et al. 2019), has been mainly measured with Joliot-type spectrometers (Viola et al. 2019; Nikkanen et al. 2020, 2024; Sellés et al. 2024). A key method for obtaining time-resolved ECS data involves administration of short (eg 300 ms) dark intervals during illumination and extracting parameters from the decay kinetics of the ECS signal during them. Fitting the dark-interval relaxation kinetics (DIRK) to a first-order function allows estimation of the *pmf* (magnitude of ECS decay), proton conductivity of the thylakoid membrane which is mainly determined by ATP synthase activity (g_H_ + parameter, calculated as the inverse of the time constant of the first-order fit), as well as proton flux (v_H_+, determined as the initial slope of ECS decay or as *pmf* × g_H_+) (Sacksteder and Kramer 2000; Cruz et al. 2004). Time-resolved ECS-DIRK measurements have also been conducted in *Synechocystis* to examine the contribution of the Mehler-like reaction to *pmf* generation (Nikkanen et al. 2020) and to study the potential role of cyanobacterial Pgr5 in downregulation of ATP synthase in high light (Nikkanen et al. 2024). A potential artefact in ECS-based measurements arises from the effect of respiratory proton pumping by NDH-1 during dark intervals. If RET is high, this will result in delayed ECS decay kinetics and underestimation of g_H_ +.

Box 2 Species specific ECS spectra ECS in many plants and green algae is typically recorded as the 550–520 nm absorbance difference (Bailleul et al. 2010), but in acidothermophilic red algae *Cyanidioschyzon merolae* and *Galdieria partita*, for example, complex ECS signals spanning 450to 546 nm comprising both linear and quadratic components were recently characterized (Wu et al. 2025). In model cyanobacterial species *Synechocystis* sp. PCC 6803 and *Synechococcus* sp. PCC 7942, ECS can be measured as the absorbance difference signal between 500 and 505 nm and between 480 and 485 nm, which correspond specifically to the ECS and correlate linearly with changes in the trans-thylakoid electric field (Viola et al. 2019). Recently, (Sellés et al. 2024) also defined ECS signals in the chlorophyll d-containing *Acaryochloris marina* as 505 to 480 nm and chlorophyll f-containing *Chroococcidiopsis thermalis* PCC 7203 as 520 to 546 nm, reflecting spectral shifts imposed by their distinct pigment compositions.

Instrumentation innovations have significantly advanced ECS-based methods. The Dual-PAM 100 spectrophotometer, equipped with a P515 module for the 550- to 520-nm range, remains widely used for plant and green algae research (Schreiber and Klughammer 2008). Attempts have been made to utilize this instrument also for ECS measurements in cyanobacteria (Zhivkovikj et al. 2025), and while a positive shift can be observed in the *Synechocystis* ECS spectrum between 510 and 550 nm, the origin of this shift remains unknown; it arises after longer exposures to experimental conditions such as hypoxia or darkness, and it does not correspond linearly to changes in membrane potential (Viola et al. 2019). The absorbance changes in this region are superimposed on top of another non-ECS signal that likely arises from a band shift of carotenoids in response to thus far unidentified changes in physiological conditions. Moreover, the observed slow kinetics of the P515 signal by Zhivkovikj et al., (2025) are not compatible with it originating from trans-thylakoid membrane potential. Conclusions from such measurements should therefore be treated with skepticism.

ECS measurements are often reported in arbitrary units, but administration of a short (optimally <1 µs) saturating single-turnover (ST) flash allows normalization of the signal to the magnitude of the ECS signal from 1 turnover of the photosystems (Mathiot and Alric 2021). ST flashes can also be used to determine the ratio of functional PSII to PSI by determining the magnitude ECS signal rise upon an ST flash after addition of hydroxylamine and DCMU to inhibit PSII charge separation and block electron transfer from Q_A_ to Q_B_, respectively. The remaining ECS signal after addition of these inhibitors thus originates from PSI turnovers only (Bailleul et al. 2010; Viola et al. 2019; Mathiot and Alric 2021; Sellés et al. 2024). A similar approach can be used to quantify cyclic electron transport (See Box 3).

Box 3 Measurement of cyclic electron transport As cyclic electron transport does not have a specific product, its reliable quantification has been a long-standing problem in photosynthesis research. Various techniques have been used to estimate CET activity, but all of them suffer from limitations, lack of specificity, or directness which should be considered. Please see the comprehensive review on CET measurement techniques by (Fan et al. 2016), which we briefly build on here in the cyanobacterial context and discuss recent method developments.

Post-illumination rise of chlorophyll fluorescence (PIFR) is often used as an indicator of PQ reduction by the NDH-1 complex (Zhang et al. 2020; Miller et al. 2022; Hubáček et al. 2024). Although the PQ pool can be reduced in the dark also by SDH and NDH-2, the phenomenon is specific to NDH-1 as demonstrated by the absence of PIFR in NDH-1 mutants (Gao et al. 2016; Zhang et al. 2020). However, the PIFR does not reflect CET activity in the light but rather NDH-1-mediated respiratory PQ reduction, which is transiently stimulated by remaining NADPH generated during illumination and by NADPH from the OPP pathway. Due to photosynthetic and respiratory electron transport occurring in the same membrane and sharing components such as the PQ pool and NDH-1, drawing a clear distinction between CET and RET is somewhat arbitrary in cyanobacteria.

Other methods include quantifying the total electron transport rate (ETR), and then repeating the experiment after applying a PSII inhibitor such as DCMU to isolate the contribution of CET. If hydroxylamine (HA) is also added to inhibit charge separation at PSII from contributing to ΔΨ, the CET rate can be derived from the post-illumination decay kinetics of the ECS signal by determining the proton flux parameter v_H_ + (as the initial slope of ECS decay) (Sacksteder and Kramer 2000; Viola et al. 2019). Acridine orange fluorescence, an indicator of pH changes (Teuber et al. 2001) was used by (Miller et al. 2021) to identify NDH-1 as being mainly responsible for CET related generation of ΔpH. Using a related approach, (Theune et al. 2021) determined initial slopes of the reduction kinetics of deconvoluted P700 and PC signals during short dark intervals to quantify the contribution of CET to total PSI ETR in cyanobacteria as up to ∼35%. These methods allow in vivo quantifications of CET that can be useful for relative comparisons between mutants or treatments, but a key limitation is the assumption that the CET rate would be the same in the presence and absence of DCMU. This is very likely not the case, as DCMU disrupts the redox poise of the PETC as well as downstream metabolism, and thereby alters the CET rate. The measured rates will also contain contribution from RET, which can be substantial in certain conditions such as mixotrophy, where reductants accumulate in the cytosol (Shimakawa, Kohara et al. 2020; Theune et al. 2021; Ortega-Martínez et al. 2024).

P700 redox kinetics during or after far-red illumination are also used to estimate CET (Gao et al. 2016; Dann and Leister 2019; Elanskaya et al. 2021). (Moore and Vermaas 2024) used sequential short actinic flashes in the presence of DCMU to fully oxidize the PQ pool in *Synechocystis* before monitoring the P700 re-reduction kinetics after the final flash. Reliability of these approaches is limited by the fact that P700 redox kinetics reflect the electron flux to all acceptors downstream of PSI. In cyanobacteria, the Mehler-like reaction has a substantial effect (Nikkanen et al. 2020; Elanskaya et al. 2021). Most importantly, however, P700 re-reduction kinetics in DCMU-treated cells in the dark are dominated by OPP-driven respiratory PQ reduction, which accounts for most of the reduction rate (Bernat et al. 2011; Kusama et al. 2022b). This makes sense, as in the dark PSI is not excited and PSI-driven “true CET” cannot occur. P700 reduction is also affected by the activity of the RTOs, FNR activity and redox state of the NADP^+^/NADPH pool, as well as the nitrogen assimilation and the HOX hydrogenase.

The ETRI-ETRII difference, based on simultaneous measurement eg using the Dual-PAM 100, has also been used to estimate the CET rate. A variation of this approach involves combining PAM fluorometry with ECS DIRK analysis to determine the *pmf*_LEF_ parameter, the fraction of *pmf* attributable to linear electron flow, as ETRII/g_H_ + (Avenson et al. 2005). This method suffers from the same limitations in accurate determination of ETRII as outlined above for PAM chlorophyll fluorometry in cyanobacteria, and from non-CET pathways affecting P700 redox kinetics. Accuracy of these parameters could be improved eg by determining ETRII directly from O_2_ evolution.

The post-illumination kinetics of the ECS signal in plants and algae have been used to estimate the partitioning of the *pmf* between the ΔpH and ΔΨ components (Cruz et al. 2001). After cessation of illumination, the ECS signal first decays at a fast rat due to relaxation of ΔΨ by efflux of positive charge through ATP synthase and can transiently undershoot the dark baseline, corresponding to the magnitude of pre-existing ΔpH. The subsequent slow recovery is attributed to slower decay of ΔpH and influx of counter-ions, mainly K^+^ (Cruz et al. 2001; Bailleul et al. 2010). Whether similar post-illumination ECS kinetics can be used to resolve *pmf* components in cyanobacteria remains unclear.

### Photoelectrochemistry

Photoelectrochemical systems have been developed for analysis of exoelectrogenesis mechanisms and electricity generation (for reviews, see Wey et al. 2019; Zhu et al. 2023). Mediator-less photoelectrochemical systems can be achieved by good electronic wiring between cells in layers on an electrode often referred to as “biofilms”. Planktonic set-ups are difficult to interpret as they require artificial mediators that have been shown to perturb photosynthesis and cell physiology (Clifford et al. 2021; Baikie et al. 2023; Yuan et al. 2025). The field would benefit from standardized protocols and integration with intracellular photosynthetic electron transfer measurements.

In two-electrode biophotovoltaics (BPVs), polarization and power curves under constant illumination measure the output of the whole biohybrid system (Bombelli et al. 2022; Howe and Bombelli 2023). Three-electrode systems use a reference electrode to precisely control the potential applied to the working electrode interfaced with cyanobacteria, allowing use of advanced (photo)electrochemical methods originally developed for redox proteins, including PSII (for reviews, see Zhang and Reisner 2019; Butt et al. 2023; Brachi et al. 2024). Cyclic voltammetry identifies redox processes at the biofilm–electrode interface and has been employed in attempts to identify the mediator(s) released from *Synechocystis* (Saper et al. 2018; Zhang et al. 2018). However, interpretation requires caution because the BG11 medium used as electrolyte contains redox active components, including manganese, that generate pseudo-photocurrents (Lai et al. 2021; Kusama et al. 2022a). Sensitive differential pulse voltammetry has been used to identify mediators secreted by heterotrophic electrogens (Marsili et al. 2008) and may identify the mediator(s) of cyanobacteria in future studies.

Chronoamperometry measures the current as it evolves from cyanobacteria in real time, whose kinetics under light/dark cycles are called the photocurrent profile. (Tanaka et al. 1988) identified a post-illumination rise in current from *Anabaena*, which they assigned to inactivation of glucose-6-dehydrogenase. *Synechocystis* and *Synechococcus* exhibit a transient decrease in current upon dark-to-light transition, which is greater for *Synechococcus*, requires an intact periplasm and is not dependent on type IV pili or extracellular polysaccharides (Wey et al. 2021, 2024). Full assignment of the photocurrent profile's features remains an important frontier, which, along with the point of exit of electrons from the PETC to exoelectrogenesis, is impeded by the lack of inhibitors that are effective, specific, and do not also act as mediators (Schneider et al. 2023).

### Electron paramagnetic resonance

Photosynthesis involves the rapid transfer of single electrons and the formation of radical ions in donor and acceptor molecules. These molecules are inherently paramagnetic, making them suitable for electron paramagnetic resonance (EPR) studies. EPR spectroscopy has been integral to several foundational discoveries in photosynthesis (Commoner et al. 1956; Golbeck et al. 1988; Sieckman et al. 1991). Recently, EPR was used to identify reversible damage of PSI iron sulfur clusters under high light and slow repair (Tiwari et al. 2016, 2024) and the unique pigments siphonein and siphonaxanthin in marine algae, which facilitate more efficient triplet-triplet energy transfer than their plant analogs (Agostini et al. 2025).

Tyrosine residues in the PSII reaction center and P700^+^ are often used to effectively quantify the ratio of functional PSI and PSII in cyanobacteria grown under different conditions using cw-EPR (Vuorijoki et al. 2017). Pulsed EPR measures the distances between paramagnetic cofactors (like donor and acceptor pairs) within the reaction centers (Bittl and Zech 2001). Spin-trapping enables the identification and quantification of highly reactive and short-lived free radicals, such as hydroxyl and superoxide radicals (Aoshima et al. 1977; Mayak et al. 1983). In cyanobacteria, direct involvement of phylloquinone in photoreduction of oxygen was suggested (Kozuleva et al. 2014). Similarly, PSI was identified as the only site in the PETC with the capacity to directly reduce molecular oxygen and accumulate hydrogen peroxide within the photosynthetic apparatus (Fitzpatrick et al. 2022). The use of nitrone-based spin traps enables the simultaneous identification of each radical species due to their ability to form distinctive spin adducts (Fitzpatrick et al. 2022). Refinement of site-directed spin labeling (SDSL) enables inter-cofactor distances to be measured with nanometer-level precision. EPR can assess membrane fluidity and molecular order within lipid bilayers using doxyl stearic acid spin probes (McConnell and Hubbell 1971). EPR employing saturation recovery directly measures the spin-lattice relaxation rate to extract dynamic and static information (Mainali et al. 2011). Furthermore, combining doxyl probes with SDSL on membrane proteins enables the simultaneous study of the effects of membrane dynamics on protein function, and vice versa (de Dios Barajas-Lopez et al. 2021).

## Concluding remarks

A major challenge in dissecting cyanobacterial electron transfer lies in the heavy reliance on chemical inhibitors, many of which have off-target or mediating activities. Non-invasive approaches, such as selective excitation with different wavelengths (Zavřel et al. 2026), will therefore be essential for future mechanistic studies. Looking ahead, a system-level strategy is needed in which complementary techniques are combined to probe multiple electron transfer reactions simultaneously within living cells. Early demonstrations, such as coupling PAM fluorometry with photoelectrochemistry (Beauzamy et al. 2020), highlight the power of integrated measurements. Finally, computational modeling of electron transport and its regulation (Höper et al. 2024; Pfennig et al. 2024) will be invaluable for unifying diverse datasets, testing mechanistic hypotheses, and predicting system behavior. Together, these advances will enable a more complete, quantitative understanding of how cyanobacteria coordinate photosynthetic, respiratory, and extracellular electron pathways.

Advances

- Ferredoxin has been identified as the electron donor to auxiliary electron transport pathways: the Mehler-like reaction as well as NDH-1
- Flavodiiron proteins are recruited to the thylakoid membrane in low ΔpH
- The NDH-1–mediated pathway has been identified as the sole major mechanism of cyclic electron transport in cyanobacteria
- Determination of the electrochromic shift in model cyanobacteria has allowed in vivo, time-resolved probing of the proton motive force and ATP synthase activity
- Analytical biofilm photoelectrochemistry can now resolve the kinetics of exoelectrogenesis, enabling the underlying bioelectrochemical mechanism to be systematically studied

Outstanding questions

- How can different biophysical methods be coupled for simultaneous measurement of different processes?
- How are the photosynthetic electron transport pathways coupled to regulation of the carbon concentrating mechanism?
- What are the specific physiological roles of the ferredoxin isoforms?
- Are the activities of NDH-1 and flavodiiron proteins or photosynthetic control (via the Rieske protein) regulated by thioredoxins or other post-translational modifications?
- How is the proton motive force and ionic environment over the thylakoid membrane regulated?
- What is the electron mediator(s) for exoelectrogenesis?
- How is the dynamic distribution of electrons to photosynthetic, auxiliary, respiratory, and extracellular electron pathways coordinated, especially under mixotrophic conditions?
- How is photosynthetic electron transport regulated in nonmodel cyanobacteria, in particular chlorophyll f-containing species?

## Data Availability

No data was used for the research described in the article.
